# *OsPT4* Contributes to Arsenate Uptake and Transport in Rice

**DOI:** 10.3389/fpls.2017.02197

**Published:** 2017-12-22

**Authors:** Ying Ye, Peng Li, Tangqian Xu, Liting Zeng, Deng Cheng, Meng Yang, Jie Luo, Xingming Lian

**Affiliations:** National Key Laboratory of Crop Genetic Improvement and National Center of Plant Gene Research, Huazhong Agricultural University, Wuhan, China

**Keywords:** *OsPT4*, rice, arsenate, phosphate transporter, uptake

## Abstract

Arsenic (As) is toxic to organisms, and elevated As accumulation in rice (*Oryza sativa*) grain may pose a significant health risk to humans. The predominant form of As in soil under aerobic conditions is As(V), which has a chemical structure similar to that of PO_4_^3-^. Rice roots take up As(V) by phosphate (Pi) transporters, such as OsPT1 and OsPT8. In the present study, we investigated the contribution of *OsPT4*, belonging to the Pht1 family, on rice As(V) uptake and transport. We determined the mRNA amounts of *OsPT*s in rice seedlings, and expressions of *OsPT1*, *OsPT4*, and *OsPT8* were up-regulated under As(V) conditions. *OsPT4*-overexpressing plants were obtained to examine the As (V) transport activity of *OsPT4* in rice. When transgenic rice grew in hydroponic culture with 25 and 50 μM As(V), the plants showed sensitivity to As(V) stress with aboveground parts showing delayed growth and the roots stunted. The *OsPT4* CRISPR lines showed the opposite phenotype. When plants were grown in 5 μM As(V) solution for 7 days, the As accumulation of *OsPT4*-overexpressing plants increased up to twice in roots and shoots. Furthermore, the arsenate uptake rates of *OsPT4*-overexpressing lines were higher compared with wild type. The *V*_max_ of As(V) uptake in *OsPT4*-overexpressing plants increased 23–45% compared with Nipponbare. In the flooded soil, the As accumulation of OsPT4-overexpressing plants increased 40–66% and 22–30% in straw and grain, respectively. While in *OsPT4*-cr plants As accumulation in roots decreased 17–30% compared with Nipponbare. Therefore, the present study indicates that *OsPT4* is involved in As(V) uptake and transport and could be a good candidate gene to generate low As-accumulating rice.

## Introduction

Inorganic arsenic (As) is a highly toxic metalloid listed as a Class-1 carcinogen by the International Agency for Research on Cancer (Smith et al., 2002). Humans ingest As unintentionally in contaminated food and drinking water. Excessive ingestion of As causes a series of acute and chronic human health problems, including skin lesions, cancers and nervous exhaustion (Anawar et al., 2002; Abernathy et al., 2003; Meharg and Rahman, 2003; Das et al., 2004). Rice (*Oryza sativa*), the most important staple food for half of the world’s people especially in South and Southeast Asia (Meharg, 2004; Alamdar et al., 2017), is a major dietary source of inorganic As because of higher As accumulation in rice than in other cereal crops (Meharg and Rahman, 2003). The As contamination in soil is made worse by non-ferrous mining, which has elevated As accumulation in rice grain up to 723 ng g^-1^ – far in excess of the Chinese maximum concentration of 200 ng g^-1^ for inorganic As in rice (Zhu et al., 2008; Williams et al., 2009; Okkenhaug et al., 2012). It is necessary to understand the mechanism of rice As accumulation and to generate low-As rice to protect human health.

Inorganic As in soil is classified into two chemical species, depending on the redox status of the soil: arsenite [As(III)] and arsenate [As(V)] (Abedin et al., 2002). As(III) is the predominant form in anaerobic paddy soil and As(V) in soil under aerobic conditions. Plant roots take up different kinds of As by different pathways. As(III) can enter root cells through nodulin26-like intrinsic proteins. Previous studies suggested that nodulin26-like intrinsic proteins are involved in As(III) transport and determine the sensitivity to As(III) stress in *Arabidopsis thaliana* (Isayenkov and Maathuis, 2008; Kamiya et al., 2009; Xu et al., 2015). In rice, the silicon transporter OsNIP2;1 (Lsi1) is functional in As(III) uptake (Zhao et al., 2010), and As(III) efflux from rice root cells to the xylem is through OsLsi2 (Ma et al., 2008; Li et al., 2009). However, when rice is grown in non-flooded soil for a long time, the soils become aerobic and then the rice roots primarily absorb As(V). In addition, because rice roots release oxygen, As(III) can be oxidized to As(V) in the rice rhizosphere (Seyfferth et al., 2010). It was reported that the chemical structure of As(V) is similar to that of PO_4_^3-^ (Das et al., 2004). In various plant species including rice, phosphorus (Pi) competes with As(V) for uptake, suggesting that they both have the same transporters (De la Rosa et al., 2006; Catarecha et al., 2007). In *Arabidopsis*, As(V) absorption is closely related to the expression of Pi transporters Pht1;1 and Pht1;4 (Shin et al., 2004; Gonzalez et al., 2005). In rice, the Pi transport pathway genes also contribute to As uptake and transport. Overexpressing the gene for the transcription factor OsPHR2 (phosphate starvation response 2) led to doubling of the As concentration in root and shoot compared with wild-type, suggesting that this gene was involved in As(V) uptake and root–shoot translocation (Wu et al., 2011). In contrast, the Pi transporter *OsPHF1* (*phosphate transporter traffic facilitator 1*) mutant lost more than half of its ability to take up As(V) (Wu et al., 2011). The Pht1 family genes (*OsPT1–OsPT1*3) in the rice genome encode Pi transporters that localize in the plasma membrane. Kamiya et al. (2013) reported that the As accumulation in rice shoots is consistent with *OsPT1* expression, indicating that OsPT1 is involved in As(V) uptake from soil to apoplast. In addition, OsPT8 was found to have a high affinity for As(V) and was a key transporter for As(V) uptake into rice roots (Wu et al., 2011; Kamiya et al., 2013). Overexpressing *OsPT8* increased the maximum As(V) influx by fivefold and mutation of *OsPT8* partially lost As(V) uptake ability (Wang et al., 2016).

After being absorbed by rice roots, As(V) is then transported into xylem vessels (Gilbert-Diamond et al., 2011). Because of its chemical structure being similar to Pi, As(V) can compete with Pi during Pi absorption and phosphorylation, forming As(V) esters and leading to imbalance in Pi metabolism (Finnegan and Chen, 2012). The As(V) esters are much less stable and hydrolyze faster than Pi esters. For example, As(V) competes with PO_4_^3-^ in ATP (adenosine triphosphate) synthesis and replaces it to form unstable adenosine diphosphate-As(V), resulting in disruption of energy flows in the cell (Hartley-Whitaker et al., 2001; Meharg and Hartley-Whitaker, 2002; Cozzolino et al., 2010). Besides, most As(V) should be reduced to As(III) inside plant cells (Su et al., 2010; Shi et al., 2016). OsACR2 has been suggested to be involved in As(V) reduction in rice (Duan et al., 2007). The latest study suggested that OsHAC1;1 and OsHAC1;2 function as As(V) reductase and were involved in the reduction of As(V) to As(III) in rice plants (Shi et al., 2016). Overexpression of OsHAC1;1 and OsHAC1;2 increased As(III) efflux and decreased As accumulation in rice shoots. The mode of action of As(III) differs from that of As(V), with As(III) acting as a cross-linking agent by binding to monothiol molecules, thiol-containing proteins and co-factors (Ha et al., 1999; Raab et al., 2005; Song et al., 2010). On the one hand, the binding of As(III) to proteins has negative effects on folding of these proteins, resulting in inactivation of many enzymes. On the other hand, As(III) complexation is the main detoxification pathway for both As(III) and As(V) (Xu et al., 2007).

Apart OsPT1 and OsPT8, it is unclear whether other Pht1 family genes are involved in As(V) uptake. The objective of the present study was to investigate the function of Pi transporter OsPT4 in rice As(V) uptake. The mRNA amount of *OsPT4* in Nipponbare was measured and *OsPT4* was induced by As(V) stress. Overexpressing *OsPT4* significantly increased the As concentration in roots and shoots, and showed higher As(V) sensitivity at high As(V) levels. This study shows that *OsPT4* plays an important role in As(V) absorption.

## Materials and Methods

### Plant Materials and Growth Conditions

Five rice (*O. sativa*) lines, the wild type Nipponbare, *OsPT2* overexpression line (*OsPT2*-ov), *OsPT4* overexpression line (*OsPT4*-ov), *OsPT4* RNA interference line (*OsPT4*-Ri), and *OsPT4* CRISPR (Clustered regularly interspaced short palindromic repeats) line (*OsPT4*-cr), were used in this study. The generation of *OsPT4*-cr is described below. *OsPT2*-ov, *OsPT4*-ov, and *OsPT4*-Ri were characterized previously (Liu et al., 2010; Ye et al., 2015).

To generate the construct of OsPT4-CRISPR vector, we designed the DNA spacer in NEB cutter^1^. The amplified PCR product including U3 promoter, spacer of OsPT4 and sgRNA (small guide RNA) were cloned into vector PJE 45/pH-Ubi-cas9-7 (Miao et al., 2013). The primers were *OsPT4*-spcer-F:5′-AGCCGGGGCTCTTGGACGCCGTTTTAGAGCTATGCTGAAA-3′, spacer-sgRNA-R: 5′-AAAAAGCAGGCTTAAAAAAAAAGCACCGACTCG- 3′, *OsPT4*-spcer-F: 5′- GGCGTCCAAGAGCCCCGGCTTGCCACGGATCATCTGCAC-3′ and Pu3-spacer-F: 5′- AGAAAGCTGGGTAAAGGGATCTTTAAACATAC GAAC-3′. The construct was transformed into Nipponbare via *Agrobacterium tumefaciens*-mediated transformation (Wu et al., 2003).

Standard rice culture solution was used in hydroponic experiments. The composition of the culture solution follows: 1.44 mM NH_4_NO_3_, 0.5 mM K_2_SO_4_, 1.0 mM CaCl_2_, 1.6 mM MgSO_4_, 0.17 mM Na_2_SiO_3_, 0.3 mM NaH_2_PO_4_, 50 μM Fe-EDTA, 0.06 μM (NH_4_)_6_Mo_7_O_24_, 15 μM H_3_BO_3_, 8 μM MnCl_2_, 0.12 μM CuSO_4_, 0.12 μM ZnSO_4_, 29 μM FeCl_3_, and 40.5 μM citric acid at pH 5.5 (Yoshida et al., 1976). The transgenic lines were grown in solution containing different concentrations of As using Na_3_AsO_4_. The solution was renewed every 5 days. The Nipponare and transgenic lines were grown in a greenhouse under 16 h/8 h, 30/22°C, day/night conditions after germination, with *c.* 60% relative humidity.

A soil experiment was performed in the experimental field in Huazhong Agricultural University, Wuhan, China. The experimental field was divided into two parts with or without Pi fertilizer. The Pi concentrations of these two fields were 15 mg kg^-1^ soil P (-P) and 30 mg kg^-1^ soil P (+P). Seedlings of Nipponbare and *OsPT4*-overexpressing plants (20-day-old) were transplanted into the soil and grown to maturity. Each treatment had 10 replicates.

### RNA Extraction and Real-Time PCR

Plant tissue samples (50–100 mg) were cut and ground with a mortar and pestle to a fine powder in liquid nitrogen. Afterward, total RNA was extracted using TRizol regent (Invitrogen, Carlsbad, CA, United States). Then, the resulted total RNAs were checked by gel electrophoresis (**Supplementary Figure S1A**). According to the manufacturer’s instructions, 3 μg of total RNA was used to synthesize the first-strand cDNA in 20 μL of reaction mixture using M-MLV reverse transcriptase (Invitrogen). Real-time PCR was performed using the SYBR Premix Ex TaqTM (TaKaRa, Shiga, Japan) with the following gene-specific primers (**Table 1**). The amplification reaction was performed on an Applied Biosystems (Foster City, CA, United States) 7500 PCR instrument. The rice *Ubiquitin 5* gene was used as the internal control.

**Table 1:** Primers for Real-time PCR.

Gene name	Forward primer/reverse primer
*OsPT1*	AGGCGGCCTACCCGAAGTAATTT/AGGCGGCCTACCCGAAGTAATTT
*OsPT2*	GCACAAACTTCCTCGGTATGCTCA/ACTCACGTCGAGACGGCATGTTTA
*OsPT3*	TGGAGGAGGTGTCCAAGGAGAA/CAATGAGCTCTGTTGAACCACCGT
*OsPT4*	GCAACGTCATCGGGTTCTTCTTCA/ACATCGTCATCGTCCTCGTTCTCG
*OsPT5*	AACTAACTCCTACAGGCAGACCGT/GAGGCAAGAATGGCAGAATGCAAC
*OsPT6*	CTGCAAACTGTACTGTAGCGCTGT/TTCGATCGATCTTCTCTGGTCTCG
*OsPT7*	AGCCGTGATCCACCCGTTAATTC/TCTCTAGTGGACTAACCACGCA
*OsPT8*	TCCAGAAGGACATCTTCACCAGCA/ATGTCGATGAGGAAGACGGTGAAC
*OsPT9*	TAAATGTTCTCATGGAGGCGGCGA/ATTGTCATAGAGACATCCGGTGCG
*OsPT10*	GTCTCCGTGTGAGTGAACTCGATCAT/CATGCACTCTCTCTGACGCACAAA
*OsPT12*	TCGTCCGGAGTTGAGATGGTGTAA/ACGCTACAAGTACGAGCTTCGCAT
*ubiquitin*	AACCAGCTGAGGCCCAAGA/ACGATTGATTTAACCAGTCCATGA

### As(V) Tolerance Assays

Rice seeds were soaked in deionized water overnight and germinated at 37°C in darkness for 3 days. Seedlings were transferred to 0.5 mM CaCl_2_ solution containing a gradient of As(V) concentrations: 0, 25, and 50 μM. Each treatment was replicated with 10 seedlings. Seedlings were grown in a controlled-environment room at 25°C constant temperature and 12-h day length. After 7 days, we photographed the growth phenotype and measured root length and shoot height.

### Determination of As Concentration

Shoots and roots were harvested separately and roots washed with distilled water before sampling. After drying at 80°C for 3 days, all samples were digested in 65% nitric acid in a MARS6 microwave (CEM) at a temperature gradient of 120–180°C for 45 min, and then diluted in deionized water. The As content of samples was determined with inductively coupled plasma-mass spectrometry (Agilent 7700 series, CA, United States).

### Measurement of Pi Concentration in Plants

Fresh samples were milled in liquid nitrogen and kept at 4°C until samples thawed. The milled samples were homogenized in 10% (w/v) perchloric acid:5% (w/v) perchloric acid (1:9) and placed on ice for 30 min. Following centrifugation at 10 000 *g* for 10 min at 4°C, the supernatant was used for Pi measurement by molybdenum blue method. The working fluid was a 6:1 ratio 0.4% (w/v) ammonium molybdate dissolved in 0.5M H_2_SO_4_ mixed with 10% ascorbic acid. Of the working fluid, 2 mL was added to 1 mL of sample solution, incubated in a water bath at 42°C for 20 min, and then cooled on ice. Sample absorbance was measured at 820 nm, and Pi concentration was calculated by normalization to fresh-weight values.

### Data Analysis

Data were examined using one-way ANOVA, followed by comparisons of means using the LSD test (Fisher’s Least Significant Difference).

## Results

### Expression Pattern of Pht1 Family Members under As(V) Conditions

**FIGURE 1:**
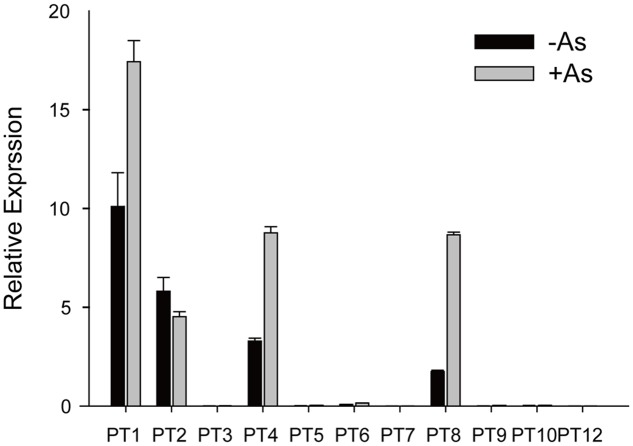
Relative expression levels of Pht1 family members in roots of Nipponbare grown in culture solution with or without 5 μM arsenate for 3 days.

As(V) is a chemical analog of Pi and can be taken up by Pi transporters, so we assayed expression levels of the Pht1 family members except for *OsPT11* and *OsPT13*, which are induced specifically during mycorrhizal symbiosis. The transcript levels of *OsPT1*, *OsPT2*, *OsPT4*, and *OsPT8* were significantly higher than that of other Pht1 family members (**Figure 1**). When rice had been grown in hydroponic conditions with 5 μM As(V) for 7 days, the expression levels of *OsPT1*, *OsPT4*, and *OsPT8* were significantly increased by 1.7, 2.7, and 5.0 times, respectively. Considering that *OsPT1* and *OsPT8* are involved in As(V) uptake and transport in rice, *OsPT2* and *OsPT4* may also have roles in As(V) uptake and transport. The *OsPT2*- and *OsPT4*-overexpressing plants were obtained via *A. tumefaciens*-mediated transformation, and expression levels of *OsPT2* and *OsPT4* were measured using real-time PCR (**Supplementary Figures S1B,C**). When transgenic plants had been grown in culture solution with 5 μM As(V) for 3 days, differences in As concentration in both shoots and roots were observed in *OsPT4*-overexpressing but not *OsPT2*-overexpressing plants (**Supplementary Figures S1D,E**). Thus, we focused on the role of *OsPT4* in As(V) uptake and transport.

### *OsPT4*-Overexpressing Rice Sensitive to As Toxicity

**FIGURE 2:**
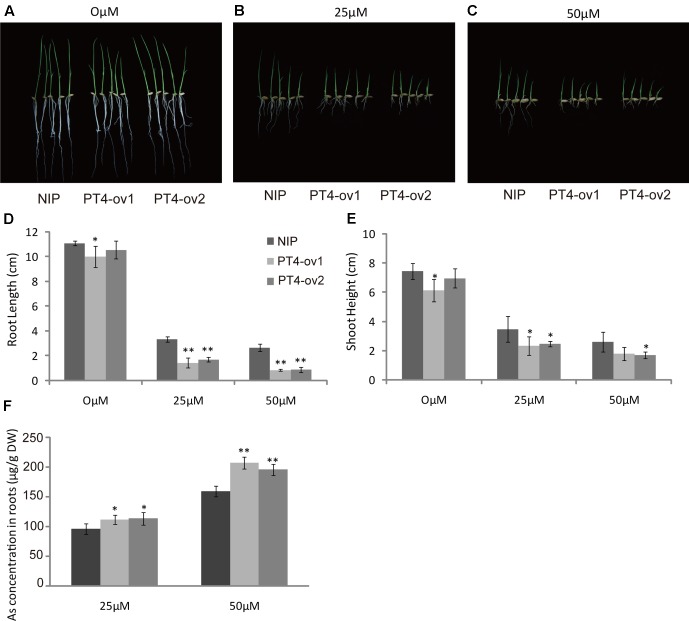
Characterization of two *OsPT4*-overexpressing plants in Nipponbare background. **(A–C)** The growth phenotype of *OsPT4*-overexpressing plants and wild-type. Plants were grown in nutrient solutions to which 0, 25, and 50 μM arsenate were added for 7 days. **(D,E)** Phenotypic analysis of OsPT4-overexpressing plants. The root length and shoot height were obtained from the 7-day-old wild-type and overexpressing plants grown in nutrient solution with different arsenate concentrations. Five plants per line were measured. **(F)** The As concentration of roots in wild-type and transgenic plants. Data are means ± SD of five biological replicates. Values are significantly different from those of wild-type: ^∗^*P* < 0.05, ^∗∗^*P* < 0.01 (one-way ANOVA). DW, dry weight.

To examine the hypothesis that *OsPT4* was involved in As(V) absorption in rice plants, a phytotoxicity experiment was performed to observe growth of *OsPT4*-overexpressing rice. The Nipponbare and *OsPT4*-overexpressing lines were seeded in hydroponic conditions with 25 or 50 μM As(V). When exposed to the As(V) condition for 7 days, rice showed an As-toxicity phenotype in which growth of aboveground parts was delayed and the roots were stunted (**Figures 2A–C**). The *OsPT4*-overexpressing plants were more sensitive to As(V) and their root length and shoot height decreased 50 and 30% compared with wild-type, respectively (**Figures 2D,E**). Furthermore, As(V) uptake by *OsPT4*-overexpressing lines and wild-type roots were determined. At both 25 and 50 μM As(V), the As concentration in roots of *OsPT4*-overexpressing rice increased 10 and 33% compared with Nipponbare (**Figure 2F**).

### The *OsPT4* Was Involved in the As(V) Uptake and Transport

**FIGURE 3:**
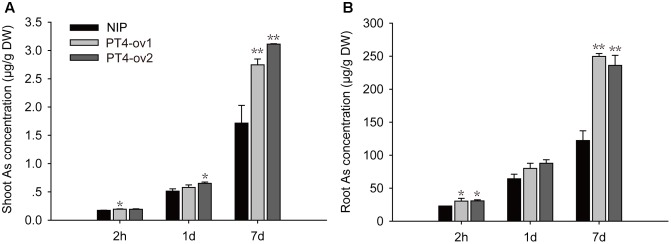
The As concentration in shoots and roots **(A,B)** of Nipponbare and *OsPT4*-overexpressing seedlings after 2 h, 1 and 7 days of exposure to 5 μM arsenate. Data are means ± SD of three biological replicates. Values are significantly different from those of wild-type: ^∗^*P* < 0.05, ^∗∗^*P* < 0.01 (one-way ANOVA). DW, dry weight.

A time-course experiment was used to investigate the ability of OsPT4 to take up As(V). The As accumulation was determined when plants were exposed to hydroponic culture with 5 μM As(V) for 2 h, 1 and 7 days. The concentration of As in rice roots and shoots increased significantly with the As(V) treatment time (**Figure 3**). When *OsPT4*-overexpressing lines grew in culture with 5 μM As(V) for 7 days, the As accumulation increased by twice in both roots and shoots.

**FIGURE 4:**
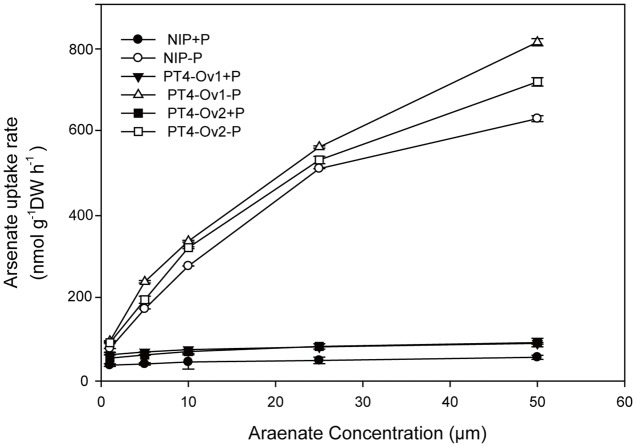
Arsenate uptake kinetics of wild type and OsPT4 overexpressing plants. Data are means ± SD of four biological replicates. DW, dry weight.

Certainly, the overexpression lines of *OsPT4* accumulated more As both in shoots and roots. It is important to determine the As uptake rate of Nipponbare and *OsPT4*-overexpressing rice. Wild type and overexpression lines were cultured in the hydroponic solution with 1–50 μM As(V) concentration under +P (100 μM) and -P (0 μM). According to the results (**Figure 4**), the As(V) uptake rate of *OsPT4* overexpression lines was higher compared with wild type under the condition with or without Pi. In the absence of Pi, the As(V) uptake kinetics could be described by a Michaelis–Menten equation (**Table 2**). The *V*_max_ (maximum influx velocity) of As(V) uptake in *OsPT4*-overexpressing plants increased 23–45% compared with Nipponbare. Additionally, the *K*_m_ values of As(V) influx in overexpression lines were 8–28% higher than that in wild type. Under the +P condition, the As(V) uptake rates of rice were significantly lower compared with plants grown in the -P condition. Moreover, As(V) uptake rate was linear over the range of As(V) concentrations tested in the solution with Pi and the slopes of *OsPT4* overexpressing plants were 1.4 to 2 times greater compared with Nipponbare. The data suggested that *OsPT4* contributes to the As(V) uptake in rice root.

**Table 2:** Fitted parameters of arsenate uptake kinetics of Nipponbare and the *PT4* overexpression line of rice.

Rice line and P treated	*V*_**max**_ (nmol g^-1^ root DW h^-1^)	*K*_**m**_ (μM)	Linear slope	*r*^2^_**adj**_
NIP+P	/	/	0.56 ± 0.12	0.950
PT4-Ov1+P	/	/	0.79 ± 0.05	0.912
PT4-Ov2+P	/	/	1.12 ± 0.21	0.906
NIP-P	625.0 ± 7.5	8.38 ± 0.3	/	0.965
PT4-Ov1-P	909.1 ± 11.2	10.72 ± 0.56	/	0.978
PT4-Ov2-P	769.2 ± 9.4	9.08 ± 0.04	/	0.955

### As(V) Concentration and Distribution in *OsPT4-*Overexpressing Rice

**FIGURE 5:**
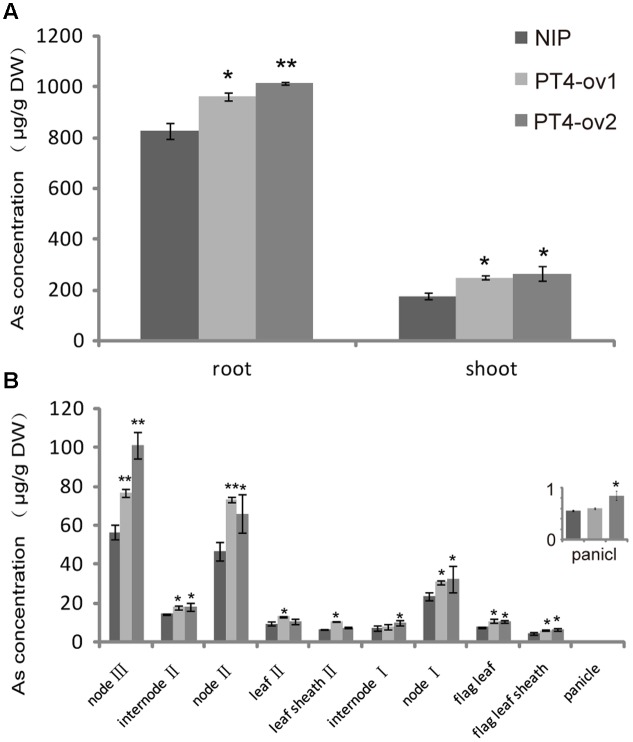
The As concentration and distribution in Nipponbare and *OsPT4*-overexpressing lines grown in hydroponic culture with 5 μM arsenate. **(A)** The As concentration of shoots and roots of Nipponbare and *OsPT4*-overexpressing lines. **(B)** The As concentration of Nipponbare and *OsPT4*-overexpressing lines in different organs of shoots. Data are means ± SD of three biological replicates. Values are significantly different from those of wild-type: ^∗^*P* < 0.05, ^∗∗^*P* < 0.01 (one-way ANOVA). DW: dry weight.

To further understand the role of *OsPT4* in As concentration and distribution in rice, the Nipponbare and *OsPT4*-overexpressing plants were grown to heading stage in hydroponic culture with 25 μM As(V). In this study, As accumulated mainly in the roots and to a lesser degree in aboveground organs (**Figure 5A**). In shoots, As was mainly in the nodes, which is the most important storage location for various metallic elements. The sum of As content in nodes accounted for 60% of total As in aboveground parts (**Figure 5B**). The As content in *OsPT4*-overexpressing lines increased in both roots and shoots by 22 and 47%, respectively. In addition, the As content of all organs in aboveground parts increased significantly, especially in nodes. The As content of the first node in *OsPT4*-overexpressing lines increased by twice that for the wild-type, while the second and third node only increased by 55 and 39% compared with wild-type, respectively. Furthermore, the As accumulation in flag leaf and flag sheath of *OsPT4*-overexpressing lines increased by 40 and 32%. The total As distribution of Nipponbare and *OsPT4*-overexpressing lines grown in the field soil was similar to that in the hydroponic experiment (**Supplementary Figure S2A**). This result was consistent with the expression pattern in different organs of rice, in which *OsPT4* was mainly expressed in root and flag leaf (**Supplementary Figure S2B**).

### Effect of Altered Expression of *OsPT4* on *OsPT1* and *OsPT8* under As(V) Conditions

**FIGURE 6:**
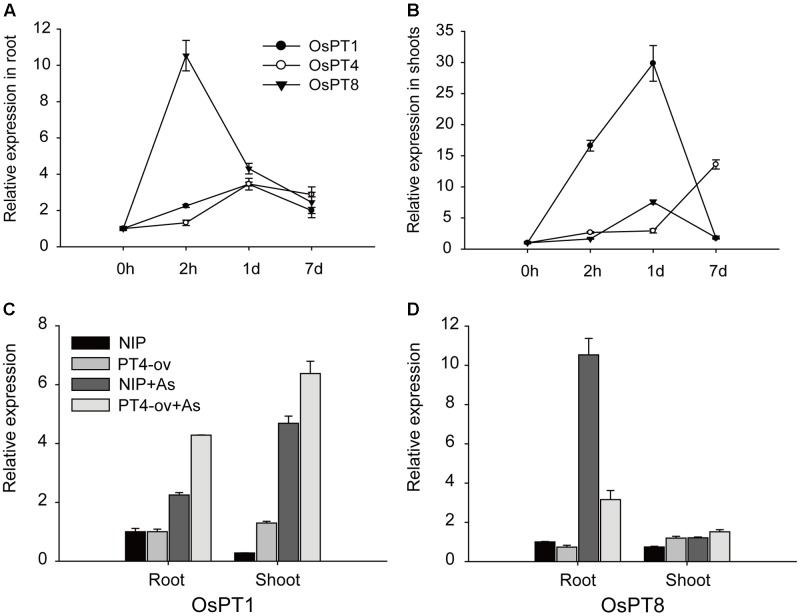
Transcript levels of *OsPT1*, *OsPT4*, and *OsPT8* in Nipponbare under arsenate stress and the effect of altered expression of *OsPT4* on *OsPT1*, *OsPT8*. **(A,B)** Expression of *OsPT1*, *OsPT4*, and *OsPT8* in roots **(A,B)** of Nipponbare seedlings grown in nutrient solutions with 5 μM arsenate after 2 h, 1 and 7 days. **(C,D)** Expression of *OsPT1* and *OsPT8* in wild type and OsPT4-overexpressing lines grown in solution with or without arsenate. Error bars indicate ±SD (*n* = 3).

Previous studies suggested that *OsPT1* and *OsPT8* were involved in As(V) uptake. The transcript levels of *OsPT1*, *OsPT4*, and *OsPT8* in rice grown in 5 μM As(V) for 2 h, 1 and 7 days were measured to determine any interaction between these genes. In roots, *OsPT8* expression rapidly increased by 10 times, then decreased and remained at a high level; however, *OsPT1* and *OsPT4* expression increased gradually and maintained a constant level until 7 days (**Figure 6A**). In the shoot, Real-time PCR analysis showed that expressions of these three genes were enhanced by As(V) (**Figure 6B**). *OsPT1* and *OsPT8* were significantly induced by 30 and 8 times, respectively, within a short period and then quickly returned to their original state. The transcript level of *OsPT4* increased gradually with treatment time, and finally increased 11 times at 7-days treatment. The induction of *OsPT1*, *OsPT4*, and *OsPT8* by As(V) raised the question of whether there was functional redundancy across the three genes. To determine this, the relative expression levels of *OsPT1* and *OsPT8* were evaluated in Nipponbare and *OsPT4*-overexpressing rice grown in hydroponic culture with or without As(V) (**Figures 6C,D**). Interestingly, expressions of *OsPT1* and *OsPT8* had no change in *OsPT4*-overexpressing rice cultured under normal conditions. The expression levels of *OsPT1* both in roots and shoots of *OsPT4*-overexpressing rice significantly increased in the As(V) condition. However, expression of *OsPT8* in *OsPT4*-overexpressing rice decreased over twofold in roots and was almost unchanged in shoots. The results suggest a lack of functional redundancy among *OsPT1*, *OsPT4* and *OsPT8*.

### Effects of *OsPT4* Overexpression on Pi and As Uptake by Rice Grown in Soil

**FIGURE 7:**
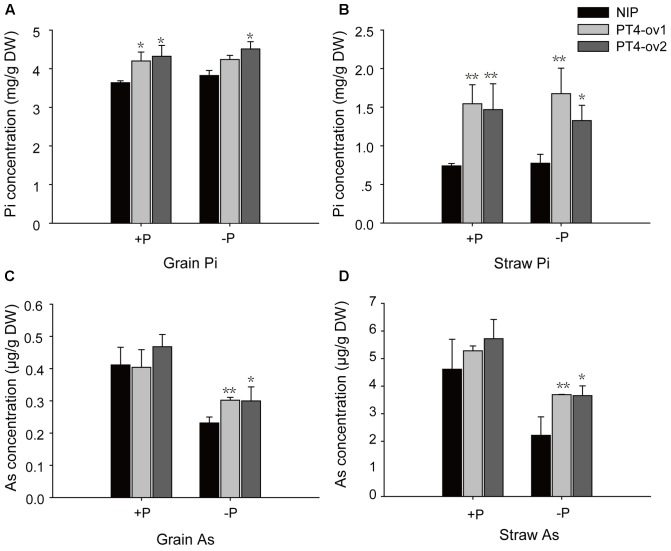
The Pi and As concentrations of wild-type and *OsPT4*-overexpressing lines in the field. The rice was grown to maturity in soil under flooded conditions including two levels of P: –P (15 mg P kg^-1^ soil) and +P (30 mg P kg^-1^ soil). **(A,B)** The Pi concentration of grain **(A)** and straw **(B)** in wild-type and *OsPT4*-overexpressing lines. **(C,D)** The As concentration of grain **(C)** and straw **(D)** in wild-type and *OsPT4*-overexpressing lines. Data are means ± SD of three biological replicates. Values are significantly different from those of wild-type: ^∗^*P* < 0.05, ^∗∗^*P* < 0.01 (one-way ANOVA). DW: dry weight.

A long-term experiment was used to investigate transgenic and wild-type plants grown to maturity in flooded soil conditions with two levels of P: -P (15 mg P kg^-1^ soil) and +P (30 mg P kg^-1^ soil). Overexpression of *OsPT4* significantly increased total Pi concentrations in both grain and straw both in -P field and +P field (**Figures 7A,B**). In -P field, *OsPT4* overexpression significantly enhanced grain and straw As accumulation by 22–30% and 40–66%, respectively, but no significant difference was shown in +P field (**Figures 7C,D**).

### Phenotypes of OsPT4 CRISPR Plants under Arsenate Stress

**FIGURE 8:**
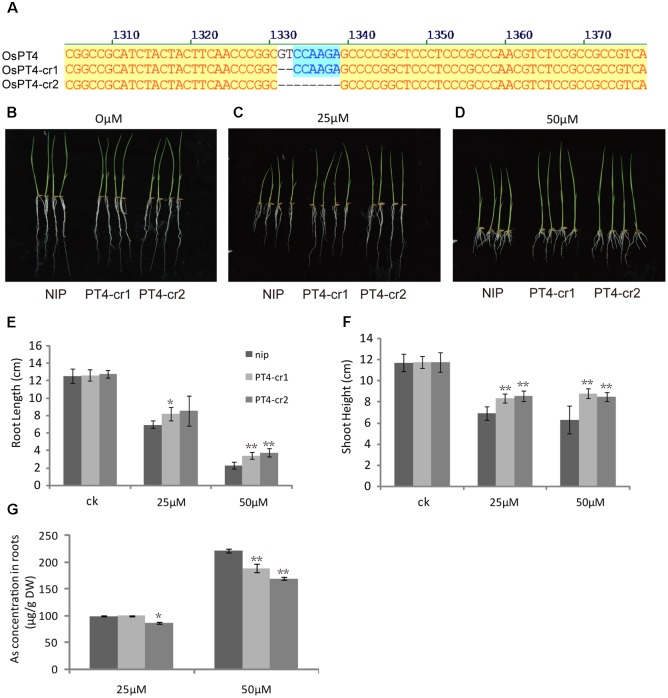
Characterization of two *OsPT4* CRISPR plants in Nipponbare background. **(A)** Detection of mutations in OsPT4. The position of target site on the gene structure is indicated. **(B–D)** The growth phenotype of *OsPT4-*cr plants and wild-type. Plants were grown in nutrient solution to which 0, 25, and 50 μM arsenate were added for 7 days. **(E,F)** Phenotypic analysis of *OsPT4* CRISPR plants. The root lengths and shoot heights were obtained from the 7-day-old wild type and *OsPT4-*cr plants grown in nutrient solution with different arsenate concentrations. Five plants per line were measured. **(G)** As concentrations of roots in wild-type and transgenic plants. Data are means ± SD of five biological replicates. Values are significantly different from those of wild-type: ^∗^*P* < 0.05, ^∗∗^*P* < 0.01 (one-way ANOVA). DW: dry weight.

In order to further study the role of *OsPT4* in rice As(V) uptake and transport, we studied the phenotype of *OsPT4*-Ri plants in solution with 0, 25, and 50 μM arsenate. The growth of *OsPT4*-Ri plants was similar to the wild type. No obvious difference in As concentration between NIP and *OsPT4*-Ri plants was observed (**Supplementary Figure S3**). Meanwhile, we obtained two different OsPT4 CRISPR lines that were treated with different As concentrations. As we expected, the *OsPT4*-cr lines showed stronger resistance to As(V) compared to wild type (**Figure 8**). The root length and shoot height of *OsPT4*-cr plants were significantly longer than wild type. Furthermore, the As accumulation of roots in *OsPT4*-cr plants decreased 17–30% compared with Nipponbare. The results obtained from *OsPT4*-cr plants confirmed that *OsPT4* is involved in As(V) uptake and transport.

## Discussion

As(V) is absorbed in roots and transported from vegetative tissues to rice grain. Elevated As accumulation in rice grain may pose a significant health risk to humans. It is important to determine how Pi transport genes contribute to As accumulation in rice. In the present study, we identified *OsPT4* as an important component of As(V) homeostasis and As tolerance in rice.

### *OsPT4* Involved in As(V) Uptake and Transport

As(V) is a toxic analog of Pi, so it can be absorbed and transported via Pi transport in plant (Xu et al., 2007). In *Arabidopsis*, previous studies suggested that AtPht1;1 and AtPht1;4 mediated a significant proportion of the As(V) uptake and a *AtPHF1* mutant was more resistant to As(V) than wild-type (Shin et al., 2004; Gonzalez et al., 2005). In rice, research has shown that Pht1 family genes participate in As(V) uptake – *OsPT1* was involved in As(V) transport from soil to apoplast and *OsPT8* functioned in As(V) uptake and resulted in a high affinity for As (Wu et al., 2011; Kamiya et al., 2013).

It was reported that the expression of *OsPT4* significantly increased in root of BRRT51 under As stress (Begum et al., 2016). In our study, the transcript levels of Pht1 family genes in Nipponbare grown in normal and As(V) conditions were measured. The expression level of *OsPT4* significantly increased in hydroponic culture containing As(V). The up-regulated expression of *OsPT4* hinted at a role in As(V) uptake. Furthermore, the ability of OsPT4 to absorb As was clearly demonstrated in the As phytotoxicity, hydroponic and field experiments. Root length and shoot height of *OsPT4*-overexpressing lines decreased 50 and 30%, respectively, compared with Nipponbare, and the As accumulation in roots of transgenic lines increased 33%. Meanwhile, the *OsPT4*-cr plants produced the opposite phenotype. Furthermore, the As(V) uptake rates of OsPT4-overexpressing plants were significantly higher than that in wild type under the growth condition with or without Pi. Differences in As concentration were also observed in grain and straw of *OsPT4*-overexpressing plants compared with wild-type in the flooded soil. All these results suggested that OsPT4 was a functional transporter in As(V) uptake.

Previous studies showed that OsPT4, a Pi-influx transporter involved in Pi acquisition and mobilization in rice, facilitates embryo development (Ye et al., 2015; Zhang et al., 2015). *OsPT4* was highly expressed in roots, specifically in the exodermis cells and cortex. Although the sclerenchymatous cells at the exodermis in rice is the first apoplastic barrier to entry of toxic As, it does not completely stop entry of As because it shares transporters with essential Pi. The strong expression of *OsPT4* in the root exodermis cells and cortex (Ye et al., 2015) may explain the high As accumulation in roots and the heightened effect of As(V) in the As phytotoxicity experiment. Overall, the results suggested that *OsPT4* was a Pi transporter sharing an ion channel with As(V) and playing an important role in As(V) uptake.

In rice, *OsPT4* is constitutively expressed in roots and shoots (Ye et al., 2015), with the highest expression in flag leaves (**Supplementary Figure S2**). However, the As concentration in roots was much higher than that in shoots (**Figure 5**). The reason for this discrepancy is likely to be due to the mitigation strategies in rice As uptake and transport. The As(V) was absorbed by *OsPT4* and the other transporters, and then quickly reduced into As(III). OsHAC1;1 and OsHAC1;2 have been reported to act as arsenate reductases in rice (Shi et al., 2016). Most of As(III) was mainly fastened in root cortex and stele, forming complex with thiol (Su et al., 2010). The uncomplexed As(III) is transported to shoots and even to grain. The formation of As(III)-thiol complex in rice roots helps to explain the reason why the As concentration in roots is four times higher than that in shoots.

The flag leaf is an essential tissue for the growth and development of rice panicles, and plays a key role in remobilization of many mineral elements from leaves to developing grain. In addition, Zhang and his team reported that *OsPT4* was involved in Pi mobilization that facilitates embryo development in rice (Zhang et al., 2015). In our work, overexpressing *OsPT4* resulted in higher As concentration than wild-type in various organs of rice shoots, such as nodes, flag leaves and panicles Thus, we deduced that *OsPT4* was probably involved in As mobilization from flag leaf to panicles and immobilization in grain.

Many researches have reported that nodes are critical hubs in controlling the distribution of mineral elements including As. Nodes have a markedly larger concentration of As than the other tissues of rice shoots (Moore et al., 2014). This was confirmed in present study, showing that the As concentration of nodes represents 60% of the total As in shoot. The possible explanation is that a large portion of the node tissues are vascular bundles and that As accumulates strongly in the phloem (Chen et al., 2015). The nodes that produce or are near crown roots may accumulate higher concentrations of As accumulation. This may explain the observation that *OsPT4*-overexpressing rice accumulated more As in node II and node III where generates crown roots. In the last few years, many mineral element transporters have been reported to function in rice nodes. A member of the rice C-type ATP-binding cassette (ABC) transporter family, *OsABCC1*, was reported as a As(III)-phytochelatin transporter. Knockout of *OsABCC1* in rice resulted in less As accumulation in the nodes and more As accumulation in the grain (Song et al., 2014). A strategy to prevent As accumulating in the grain is accumulate As in the nodes, especially those close to crown roots.

### Interaction among Pht1 Family Proteins in As(V) Uptake

There are a reported 13 members of the rice Pht1 family and most share a similar protein structure and the same protein destination (localized to the plasma membrane). Among their encoding genes, *OsPT1*, *OsPT4*, and *OsPT8* were induced by As(V) stress. Individually overexpressing these three genes led to higher As accumulation than their background. These results raised the question of whether there is a network among these three Pi transporters playing specific and/or overlapping roles in As accumulation in rice.

The sensitivity to As(V) stress of *OsPT4*-overexpressing plants and *OsPT8* mutants, assessed in As phytotoxicity experiments, showed that both *OsPT4* and *OsPT8* were involved in As(V) uptake from roots (Wang et al., 2016) (**Figure 2**). Analyses of the GUS reporter gene driven by the promoters of *OsPT4* and *OsPT8* indicated a partial overlap in their spatial expression patterns, because the strongest expression of *OsPT4* and *OsPT8* were in the epidermis and cortex (Jia et al., 2011; Ye et al., 2015). Additionally, real-time PCR analysis revealed the same expression pattern of *OsPT4* and *OsPT8* in BRRI33 and BRRI51 under As stress along with lower regulation in BRRI33 and higher regulation in BRRI51 (Begum et al., 2016). And both *OsPT4* and *OsPT8* were up-regulated in overexpression lines of *OsPAP21b* and *OsHAD1* under Pi-deficient conditions (Mehra et al., 2017; Pandey et al., 2017). Moreover, the transcript level of *OsPT8* in roots was dramatically attenuated in *OsPT4*-overexpressing plants grown in As(V) conditions (**Figure 6D**). These results indicated that there may be a functional overlap of OsPT4 with OsPT8. However, contrary to this assumption, *ospt8* mutants lost almost half of their As(V) uptake ability when seedlings were exposed to 1–2 μM As(V)(Wang et al., 2016). In our study, OsPT4-cr lines displayed significantly lower As concentrations in roots. The similar phenomenon has been observed in *ospt4* mutant (Cao et al., 2017). The attenuation of function of *OsPT8* was not compensated for by *OsPT4*, and so *OsPT4* and *OsPT8* were non-redundant in As(V) uptake. Furthermore, the expression variance of *OsPT4* and *OsPT8* in the time-course experiment also differed. In the present study, *OsPT8* expression in rice roots quickly increased by 10 times when plants were treated with As(V) for 2 h and then dropped (**Figure 6A**). Over the same time periods, the transcript level of *OsPT4* increased gradually and remained at a high level. In flooded soil, overexpressing *OsPT8* did not change the As accumulation in straw and grain (Wu et al., 2011), but the As accumulation in *OsPT4*-overexpressing plants increased in Pi-replete conditions (**Figures 7C,D**). These results strongly suggest that *OsPT4* and *OsPT8* had similar expression patterns but different regulation pathways in As uptake.

In *Arabidopsis*, there was a possible functional overlap of Pht1;1 with Pht1;4. In the As(V) condition, the double mutant showed a more resistant phenotype compared with background and the mutants for single genes (Shin et al., 2004). In rice, the promoters of *OsPT1* and *OsPT4*, unlike *OsPT8*, did not contain the P1BS element. The expression level of *OsPT1* and *OsPT4* was not effect by Pi supply conditions (Wu et al., 2013). Kamiya et al. (2013) reported that *OsPT1* was involved in As accumulation in shoots, a conclusion consistent with our result that the expression of *OsPT1* in shoots rapidly increased by 30 times (**Figure 6B**). The As accumulation in shoots of *ospt1* mutant was not significantly different to its background in Pi-replete condition (Kamiya et al., 2013), indicating that there may be a functional overlap between *OsPT1* and *OsPT4*. We also found that altered expression of *OsPT4* did affect expression of *OsPT1*. The transcript level of *OsPT1* in rice roots and shoots dramatically increased in *OsPT4*-overexpressing plants grown in normal and As(V) conditions (**Figure 6C**). Therefore, it was logical to assume that *OsPT1* and *OsPT4* shared a similar or the same pathway in rice As(V) uptake and translocation. Studies utilizing double mutants of these two genes are needed to test this hypothesis.

### *OsPT4* Could Be a Candidate Gene in Rice Breeding

Although there were significant increases of As concentration in *OsPT4*-overexpressing plants grown in hydroponic solution with Pi supplement, differences in As concentration in grain and straw were observed under -P but not +P flooded soil (**Figure 7**). The latest study showed that knockout of *OsPT4* could significantly decrease the inorganic As in rice grain (Cao et al., 2017). Since inorganic As is classified as Class-1 carcinogen, decreasing the concentration of inorganic As in rice grain is an important rice breeding target to protect human health. Under these circumstances, *OsPT4* which is involved in Pi and As uptake is a candidate gene to generate a high Pi-efficiency and low As-accumulating rice.

## Author Contributions

Experimental design: XL and YY. Experiments: YY, PL, TX, LZ, JL, and DC. Data analysis: YY and MY. Manuscript preparation: XL and YY. Supervision, funding and reagents: XL.

## Conflict of Interest Statement

The authors declare that the research was conducted in the absence of any commercial or financial relationships that could be construed as a potential conflict of interest.

## Footnotes

1 http://tools.neb.com/NEBcutter2/

## Supplementary Material

FIGURE S1 The characteristics of *OsPT2*- and *OsPT4*-overexpressing plants. The expression levels and As contents of *OsPT2* and *OsPT4* were determined with real-time polymerase chain reaction and inductively coupled plasma mass spectrometry (ICP-MS), respectively. **(A)** Total RNA of wild type, *OsPT2*- and *OsPT4*-overexpressing plants. **(B)** Relative expression levels of *OsPT2* in *OsPT2*-overexpressing plants compared with background. **(C)** Relative expression levels of *OsPT4* in *OsPT4*-overexpressing plants compared with background. **(D,E)** The As accumulation in shoot **(D)** and root **(E)** of wild-type and transgenic plants after exposure to 5 μM arsenate for 7 days. Data are means ± SD of three biological replicates. Values are significantly different from those of wild-type: ^∗^*P* < 0.05, ^∗∗^*P* < 0.01 (one-way ANOVA). DW: dry weight.

FIGURE S2 The As concentration in *OsPT4*-overexpressing plants and the expression pattern of *OsPT4* in Nipponbare. **(A)** The As concentration of wild-type and *OsPT4*-overexpressing plants grown to heading stage in flooded soil. Data are means ± SD of three biological replicates. Values are significantly different from those of wild-type: ^∗^*P* < 0.05, ^∗∗^*P* < 0.01 (one-way ANOVA). DW: dry weight. **(B)** The relative expression levels of *OsPT4* in different organs of Nipponbare. Error bars indicate ± SD (*n* = 3).

FIGURE S3 Phenotypes of OsPT4 RNA interference plants. **(A–C)** The growth phenotype of OsPT4-Ri plants and wild type. Plants were grown in nutrient solution to which 0, 25, and 50 μM arsenate were added for 7 days. **(D)** As concentrations of roots in wild-type and OsPT4-Ri plants. Data are means ± SD of five biological replicates. Values are significantly different from those of wild-type: ^∗^*P* < 0.05, ^∗∗^*P* < 0.01 (one-way ANOVA). DW, dry weight.
