# Supplementary figures and images for: OsPT4 Contributes to Arsenate Uptake and Transport in Rice
Source: Front Plant Sci. 2017 Dec 22;8:2197. doi: 10.3389/fpls.2017.02197 (PMC5744437; doi:10.3389/fpls.2017.02197)

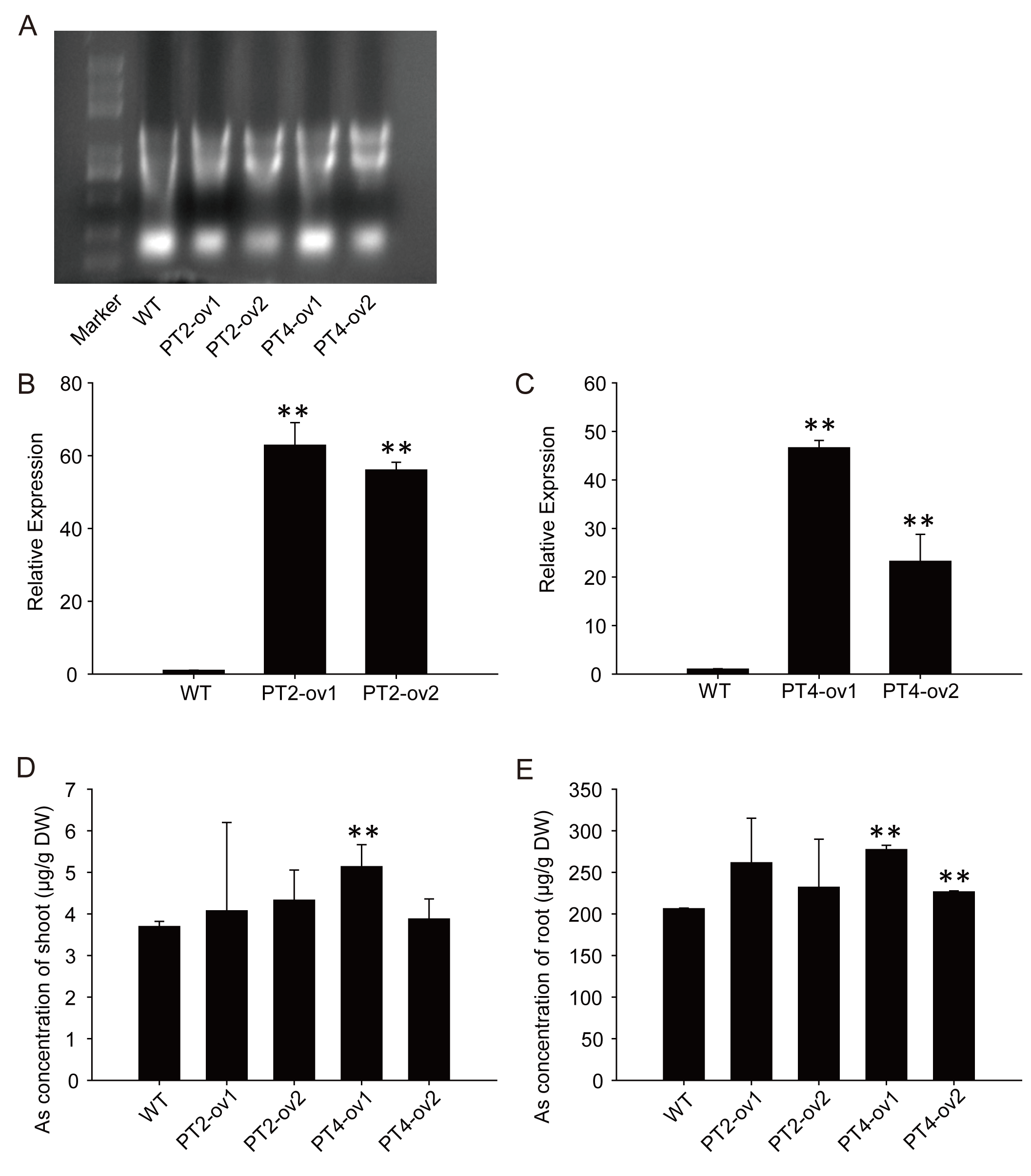

Supplement: FIGURE S1 — The characteristics of OsPT2- and OsPT4-overexpressing plants. The expression levels and As contents of OsPT2 and OsPT4 were determined with real-time polymerase chain reaction and inductively coupled plasma mass spectrometry (ICP-MS), respectively. (A) Total RNA of wild type, OsPT2- and OsPT4-overexpressing plants. (B) Relative expression levels of OsPT2 in OsPT2-overexpressing plants compared with background. (C) Relative expression levels of OsPT4 in OsPT4-overexpressing plants compared with background. (D,E) The As accumulation in shoot (D) and root (E) of wild-type and transgenic plants after exposure to 5 μM arsenate for 7 days. Data are means ± SD of three biological replicates. Values are significantly different from those of wild-type: ∗P < 0.05, ∗∗P < 0.01 (one-way ANOVA). DW: dry weight. [file Image_1.TIF]

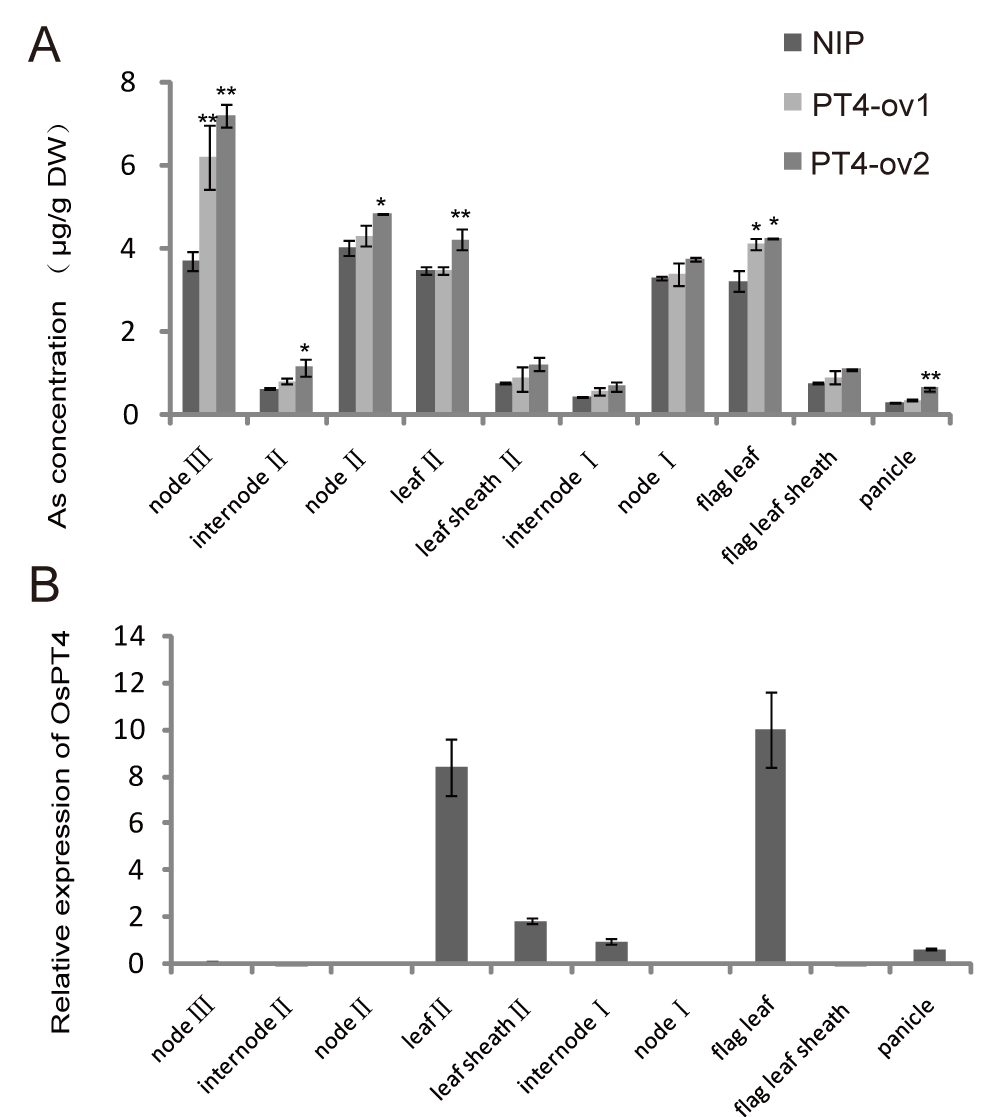

Supplement: FIGURE S2 — The As concentration in OsPT4-overexpressing plants and the expression pattern of OsPT4 in Nipponbare. (A) The As concentration of wild-type and OsPT4-overexpressing plants grown to heading stage in flooded soil. Data are means ± SD of three biological replicates. Values are significantly different from those of wild-type: ∗P < 0.05, ∗∗P < 0.01 (one-way ANOVA). DW: dry weight. (B) The relative expression levels of OsPT4 in different organs of Nipponbare. Error bars indicate ± SD (n = 3). [file Image_2.TIF]

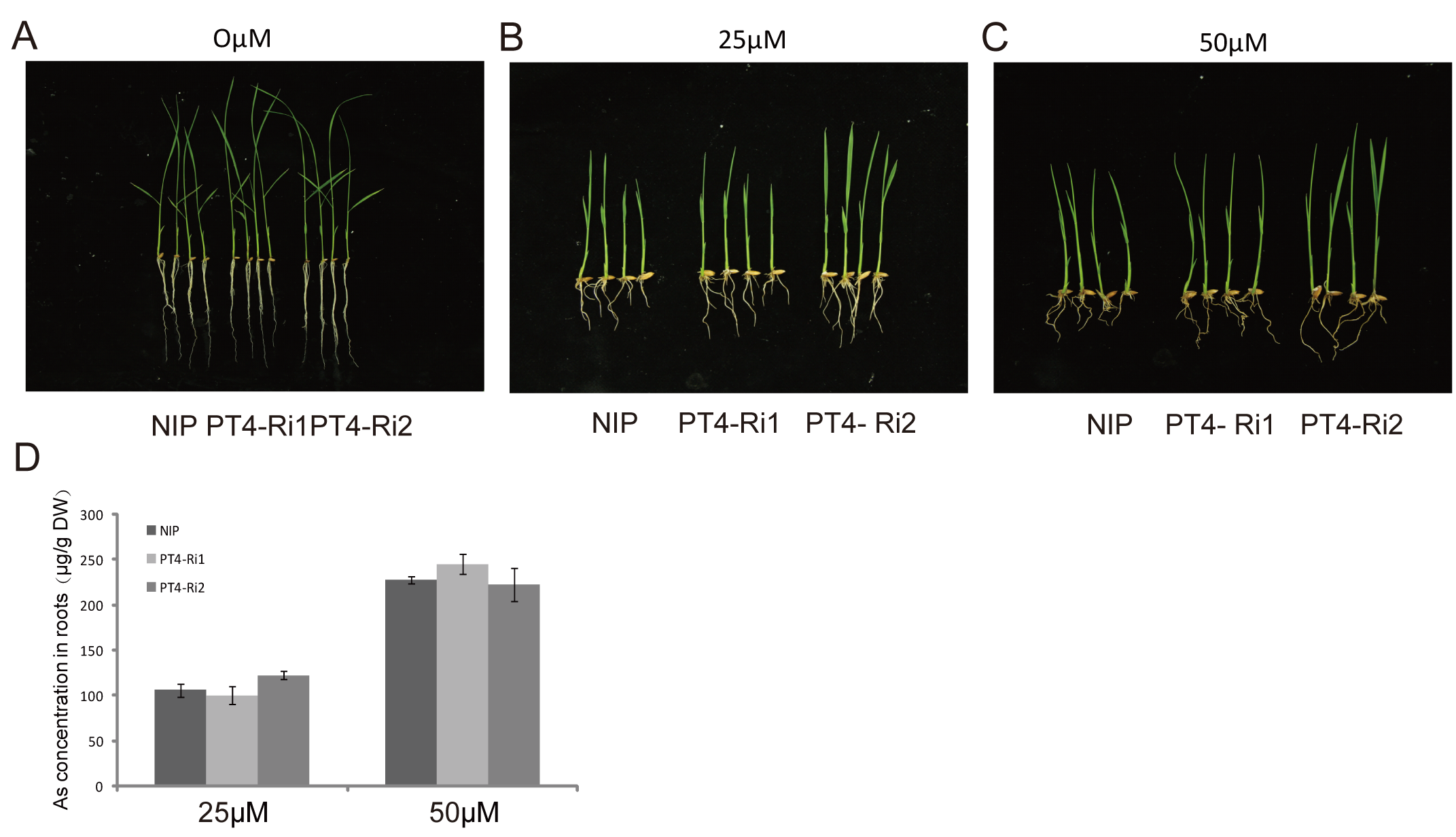

Supplement: FIGURE S3 — Phenotypes of OsPT4 RNA interference plants. (A–C) The growth phenotype of OsPT4-Ri plants and wild type. Plants were grown in nutrient solution to which 0, 25, and 50 μM arsenate were added for 7 days. (D) As concentrations of roots in wild-type and OsPT4-Ri plants. Data are means ± SD of five biological replicates. Values are significantly different from those of wild-type: ∗P < 0.05, ∗∗P < 0.01 (one-way ANOVA). DW, dry weight. [file Image_3.TIF]
